# Effects of continuous vs interval exercise training on oxygen uptake efficiency slope in patients with coronary artery disease

**DOI:** 10.1590/1414-431X20154890

**Published:** 2016-02-05

**Authors:** D.M.L. Prado, E.A. Rocco, A.G. Silva, D.F. Rocco, M.T. Pacheco, P.F. Silva, V. Furlan

**Affiliations:** 1 Grupo TotalCare, Amil, São Paulo, SP, Brasil; 2 Universidade Santa Cecília, Santos, SP, Brasil

**Keywords:** Coronary artery disease, Exercise, Cardiorespiratory fitness, Ventilatory efficiency

## Abstract

The oxygen uptake efficiency slope (OUES) is a submaximal index incorporating cardiovascular, peripheral, and pulmonary factors that determine the ventilatory response to exercise. The purpose of this study was to evaluate the effects of continuous exercise training and interval exercise training on the OUES in patients with coronary artery disease. Thirty-five patients (59.3±1.8 years old; 28 men, 7 women) with coronary artery disease were randomly divided into two groups: continuous exercise training (n=18) and interval exercise training (n=17). All patients performed graded exercise tests with respiratory gas analysis before and 3 months after the exercise-training program to determine ventilatory anaerobic threshold (VAT), respiratory compensation point, and peak oxygen consumption (peak VO_2_). The OUES was assessed based on data from the second minute of exercise until exhaustion by calculating the slope of the linear relation between oxygen uptake and the logarithm of total ventilation. After the interventions, both groups showed increased aerobic fitness (P<0.05). In addition, both the continuous exercise and interval exercise training groups demonstrated an increase in OUES (P<0.05). Significant associations were observed in both groups: 1) continuous exercise training (OUES and peak VO_2_ r=0.57; OUES and VO_2_ VAT r=0.57); 2) interval exercise training (OUES and peak VO_2_ r=0.80; OUES and VO_2_ VAT r=0.67). Continuous and interval exercise training resulted in a similar increase in OUES among patients with coronary artery disease. These findings suggest that improvements in OUES among CAD patients after aerobic exercise training may be dependent on peripheral and central mechanisms.

## Introduction

Cardiopulmonary exercise testing (CPX) is a highly reliable and well-validated approach to assessing aerobic performance and monitoring exercise tolerance in patients with cardiovascular disease (1). In this context, Baba et al. (2) introduced the oxygen uptake efficiency slope (OUES), an objective and reproducible measure of cardiopulmonary function. The OUES is derived from the single regression analysis between oxygen uptake and minute ventilation during incremental exercise. Importantly, OUES evaluates the functional capacities of several organ systems, such as cardiovascular, pulmonary and skeletal muscle metabolism during exercise (2,3) in a single index. The OUES has been investigated as an index of cardiopulmonary functional reserve in patients with various conditions (4 5 6). Of particular note, a study that followed patients with cardiovascular disease over 6 years showed that the OUES was a good prognostic indicator (7).

Aerobic exercise training has been recommended as a non-pharmacological treatment for patients with coronary artery disease (CAD) (8,9). In this regard, continuous exercise training (CET) has been shown to promote increased cardiorespiratory fitness in CAD patients (10). However, over the last few decades, there has been increasing interest in interval exercise training (IET) for cardiac rehabilitation (10,11). IET consists of periods of high-intensity exercise alternated with periods at lower intensity; this allows cardiac patients to complete short exercise bouts at a higher intensity than would have been possible during continuous exercise. Previous investigations (11,12 [Bibr B13]) have also shown that IET effectively improves the aerobic fitness of CAD patients.

Although both CET and IET have been shown to improve aerobic fitness, there is little information about the effects of the mode and intensity of exercise training on OUES in CAD patients. Thus, the purpose of this study was to evaluate the effects of CET and IET on OUES in patients with CAD.

## Subjects and Methods

### Population

The participants were patients admitted to the coronary care unit of the TotalCor Hospital for the diagnosis of CAD. After being discharged from the hospital, the patients enrolled in a cardiac rehabilitation program at the cardiorespiratory rehabilitation center of the Amil group. Thirty-five CAD patients (59.3±1.8 years old; 28 men, 7 women) were randomly divided into two groups: CET (n=17) and IET (n=18). This trial was designed to test the exercise-training modalities for noninferiority, with the key secondary objective of testing for superiority with respect to the OUES. The inclusion criterion was having stable CAD diagnosed by coronary angiography. Exclusion criteria included having unstable angina pectoris, complex ventricular arrhythmias, pulmonary congestion, and orthopedic or neurological limitations to exercise. Patients remained on their standard medications throughout the study, and no changes in medications were reported. The study was approved by the Ethics Committee of the Universidade Santa Cecilia (66/2011) and all of the study participants gave their written informed consent.

### Graded exercise test

Maximal graded exercise tests were carried out on a programmable treadmill (DigiStress model pulsar, Brazil) before, and three months after commencing the IET or CET intervention. Gas exchange and ventilatory variables were measured continuously during the gas exchange tests, breath-by-breath, using an open-circuit spirometry procedure on an exercise-based system (SensorMedics, model Vmax 229 Pulmonary Function/Cardiopulmonary Exercise Testing Instrument, USA). The following variables were obtained breath-by-breath and expressed as 30-s averages: pulmonary oxygen uptake (VO_2_ mL·kg^-1^·min^-1^, standard temperature and pressure, dry) pulmonary ventilation (VE L/min body temperature and pressure, saturated), end-tidal carbon dioxide pressure (PetCO_2_ mmHg), ventilatory equivalent ratio for oxygen (VE/VO_2_), and ventilatory equivalent ratio for carbon dioxide (VE/VCO_2_). Before each test, the gas analyzers were calibrated using a gas mixture containing known concentrations of carbon dioxide and oxygen balanced with nitrogen, and the flow meter was calibrated using a 3-L syringe. Heart rate was continuously recorded at rest, during the graded exercise testing, and during the recovery period using a 12-lead ECG (HW Systems, HeartWare Ltda, USA). All tests in this study were performed in the same laboratory at a room temperature of 20-23°C.

The subjects performed a ramp-like progressive exercise test to exhaustion on the treadmill. The exercise workload (speed and/or slope) was increased every 1 minute with completion of the incremental part of the exercise test occurring between 8 and 12 min. The following criteria were used to define maximal effort: 1) participants demonstrated subjective evidence of exhaustion (unsteady gait, facial flushing, and hyperpnea), and either 2) peak heart rate (HR) ≥95% age-predicted maximum, or 3) maximal respiratory exchange ratio (RER) ≥1.10 (1).

### Ventilatory anaerobic threshold

The ventilatory anaerobic threshold (VAT) was defined as the break point between the increase in the carbon dioxide output and VO_2_ (V-Slope) or the point at which VE/VO_2_ reached its minimum value and began to rise without a concomitant rise in VE/VCO_2_ (13).

### Respiratory compensation point

The respiratory compensation point (RCP) was defined as the point at which VE/VCO_2_ reached its minimum value and began to rise, and the highest value of PetCO_2_ before its progressive fall (14). In the present investigation, all patients reached the RCP.

### Peak oxygen consumption

The peak oxygen consumption was defined as the maximum VO_2_ attained during the exercise period in which the subject reached exhaustion (analog scale from Borg's scale of perceived exertion).

### Oxygen uptake efficiency slope calculation

The OUES was assessed based on the respiratory data during exercise by calculating the slope of the linear relationship between VO_2_ (y-axis) and the logarithm of VE (x-axis) using single regression analysis. The OUES was derived from the relationship: VO_2_=a log10 VE+b, where a is the OUES and b is the intercept. Before inclusion in the regression analysis, respiratory data were averaged every 30 s from the beginning of the second minute of exercise until evident exhaustion. The first minute of exercise was not included in the analyses because of irregular breathing patterns during early exercise (2).

### Ventilatory efficiency analysis

The VE/VCO_2_ Slope, which reflects the rate of increase in minute ventilation per unit increase in CO_2_ production, was obtained by linear regression analysis of the relationship between VE and VCO_2_ during exercise using the data from the entire exercise test excluding the first minute (15).

The difference in end-tidal carbon dioxide pressure from at rest to its highest value during exercise (Δ PeTCO_2_ rest-exercise) was also analyzed (1).

### Exercise-training program

A supervised exercise-training program was conducted at the cardiorespiratory rehabilitation center of the Amil Group. The exercise-training program consisted of three 60-min exercise sessions per week for a 3-month period. Each exercise session included a 5-min warm-up, 42 or 50 min of aerobic exercise, and 5 min of cool-down exercises. The CET consisted of 50-min of treadmill exercise at VAT intensity. The IET consisted of seven sets of 3 min at RCP and seven sets of 3 min of exercise at VAT intensity, totaling 42 min of exercise. The estimated energy expenditure for both exercise-training modalities ranged from 200 to 230 kcal/min. Heart rate was monitored throughout the sessions to ensure that all patients exercised within the intensity limits. Adherence to the exercise-training program was assessed based on the percentage of exercise sessions attended, with all patients attending more than 80% of the sessions.

### Statistical analysis

The statistical analyses were carried out using SPSS version 16.0 (SPSS Inc., USA). Data are reported as means±SE. A P value of <0.05 was considered to be statistically significant. The normality of distribution was checked for all variables using the Kolmogorov-Smirnov test. Student's unpaired *t*-tests and Fischer's exact tests were used to assess baseline differences between the CET and IET groups for all dependent variables.

Two-way analysis of variance (ANOVA) with repeated measures was performed to test for possible within-group and between-group differences in physical characteristics, cardiorespiratory parameters, and the OUES. When significant differences were detected, Tukey’s *post hoc* comparisons were performed. Single regression analysis was used to investigate the relationship between OUES and cardiorespiratory parameters after exercise training.

For the CET group, the sample size of 18 provided the power (96%) to detect a difference in OUES based on a relevant difference of 0.32 L/min, an SD of 0.35 L/min, α=0.05, and a two-tailed test of significance. For the IET group, the sample size of 17 provided the power (99%) to detect a difference in OUES based on a relevant difference of 0.32 L/min, an SD of 0.30 L/min, α=0.05, and a two-tailed test of significance.

## Results

### Effects of interventions

Before the interventions, there were no between-group differences in physical characteristics (Table 1). After the interventions, neither the CET nor IET group showed any reduction in body weight (Table 2).

**Table:**
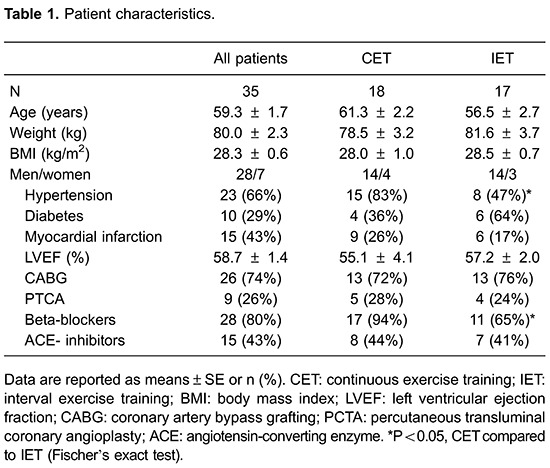

**Table:**
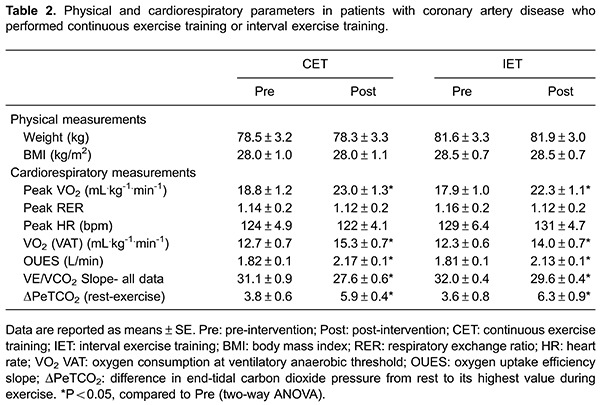

With respect to clinical parameters, the CET group had more patients with hypertension than the IET group; however, no between-group differences in left ventricular ejection fraction were observed (Table 1). More patients in the CET group were being treated with beta-blockers than in the IET group.

Both groups had similar levels of aerobic fitness (Table 2). In addition, no statistically significant differences in pre-intervention OUES were present (Table 2).

### Cardiorespiratory response

After the interventions, CAD patients subjected to either CET or IET showed no differences in peak RER or HR. However, both the CET and IET groups showed an increase in VO_2_ at VAT and peak VO_2_. Between-group comparisons revealed similar increases in aerobic fitness after the exercise programs (Table 2). The OUES values were similarly and significantly increased from baseline in both the CET and IET groups after the exercise-training program (Table 2).

In addition to ventilatory efficiency, CAD patients had lower values for VE/VCO_2_ Slope after either CET or IET than at baseline. In addition, after the interventions both groups demonstrated an increase in ΔPeTCO_2_ at rest (Table 2). The increase in ventilatory efficiency after the interventions was similar between groups (Table 2).

**Figure 1:**
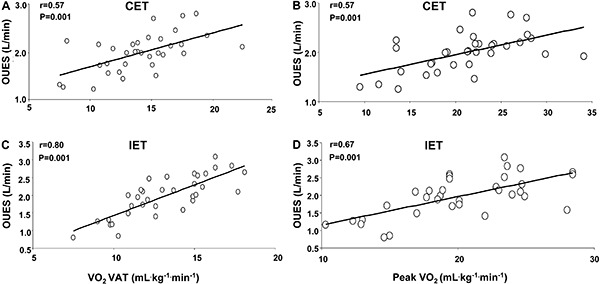
Relationship between oxygen uptake efficiency slope (OUES) and aerobic fitness in patients with coronary artery disease subjected to continuous exercise training (CET; *A* and *B*) and interval exercise training (IET; *C* and *D*). VO_2_ VAT: oxygen consumption at ventilatory anaerobic threshold; peak VO_2_: peak oxygen consumption.

The results of the correlation analysis between OUES and aerobic fitness after either CET or IET are shown in Figure 1. Significant associations were observed in both groups: 1) CET (OUES and VO_2_VAT r=0.57, P=0.001; OUES and Peak VO_2_ r=0.57, P=0.001); 2) IET (OUES and VO_2_VAT r=0.80, P=0.001; OUES and Peak VO_2_ r=0.67, P=0.001). However, the OUES was not significantly correlated with ventilatory efficiency in either of the groups: CET (OUES and VE/VCO_2_ Slope r=-0.16, P=0.33); IET (OUES and VE/VCO_2_ Slope r=-0.19, P=0.29).

**Figure 2:**
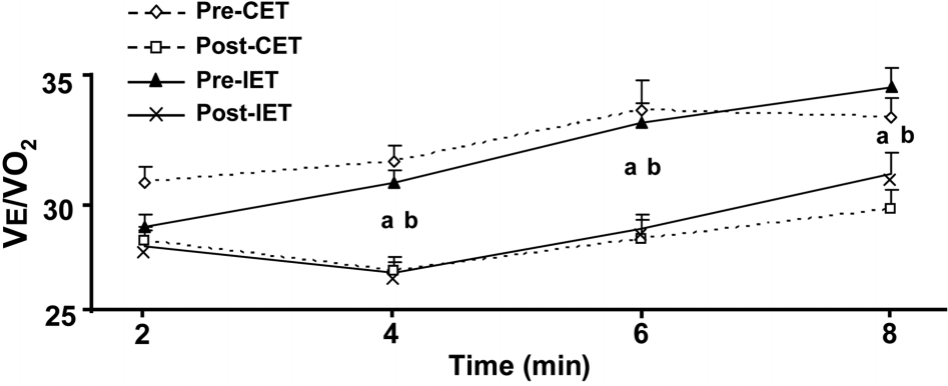
Ventilatory equivalent ratios for oxygen (VE/VO_2_) at baseline (Pre), and after 3 months (Post) of continuous exercise training (CET) and interval exercise training (IET), in patients with coronary artery disease. ^a^P<0.05, compared to pre-CET; ^b^P*<*0.05, compared to pre-IET (two-way ANOVA).

After the interventions, both groups showed a reduction in ventilatory equivalent ratios for oxygen at matched work rates throughout the graded exercise test (Figure 2).

## Discussion

The main findings of this study were: 1) CET and IET increased OUES and aerobic fitness in CAD patients; 2) CET and IET resulted in the same degree of increase in OUES; 3) significant associations were observed between OUES and aerobic capacity for CET and IET.

This study evaluated the effects of interval and continuous exercise on OUES in patients with CAD. Determining the mechanisms involved in increasing OUES after exercise training are beyond the scope of the present study; however, we suggest that factors related to peripheral metabolic adaptations, such as increased aerobic metabolism, may be involved. After 12 weeks of either CET or IET, CAD patients demonstrated similar increases in VO_2_ at VAT. In addition, our results showed significant post-intervention associations between OUES and VO_2_ at VAT.

Baba et al. (2) proposed that the physiological mechanisms reflected in the OUES are the development of metabolic acidosis, which is controlled by distribution of blood to skeletal muscles, and also the physiologic dead space, which is affected by perfusion of the lungs. Thus, an increase in OUES after aerobic exercise training indicates that a given oxygen uptake has been achieved with lower ventilatory demand at submaximal workloads (16). Our results showed that the ratio of VE to VO_2_ (reflecting the ventilatory requirement for a given amount of work) was significantly reduced at matched work rates after CET and IET. Notably, an increase in capillary density, muscle blood flow, and mitochondrial density after aerobic exercise training leads to a delay in the onset of metabolic acidosis, thereby decreasing the ventilatory response during exercise. These results are in accordance with previous investigations demonstrating the effects of exercise training on OUES in cardiac patients (6,16). For instance, Van Laethem et al. (6) showed an increase in OUES after a 6-month exercise-training program in patients with chronic heart failure. Furthermore, in the same study, the authors observed that the increase in OUES correlated with improvement in VAT.

In the present study, after a 3-month exercise intervention, both training methods were found to result in the same level of improvement in aerobic fitness. These findings agree with those of previous studies that have demonstrated similar effectiveness of CET and IET for increasing cardiorespiratory fitness in CAD patients (17,18). However, other studies (11,12) have observed a greater increase in fitness in response to IET than to CET. Rognmo et al. (11) reported a greater increase in cardiorespiratory fitness among CAD patients who performed IET, alternating between 4 min at higher intensity (80%-90% peak VO_2_) and 3 min at moderate intensity (50%-60% peak VO_2_) throughout the exercise session. In the same investigation (11), the CAD patients who performed CET completed 41 min of moderate intensity (50%-60% peak VO_2_) exercise for a 10-week period. In contrast, in the present study the CAD patients in the IET group performed seven 3-min sets of exercise at RCP (80%-90% peak VO_2_) and seven 3-min sets of exercise at moderate intensity corresponding to the VAT, whereas the CET group patients performed 50 min of exercise at VAT (70%-80% peak VO_2_), for 12 weeks. We suggest that the similar fitness improvements observed in both groups may be attributed to the greater relative intensity and higher volume of exercise performed by the CET group in our present study. Controversy remains regarding the mode and intensity of aerobic exercise that yields optimal cardiorespiratory effects in CAD patients.

In this investigation, the improvement in OUES after aerobic exercise training was positively correlated with peak VO_2_. Similarly, Defoor et al. (16) showed an improvement in OUES in 425 patients with CAD after an exercise-training program. In the same study, the authors observed similar increases in both peak VO_2_ and OUES. These findings indicate that the OUES may be a sensitive measure for quantifying exercise performance improvements in cardiac patients.

It is important to mention that a previous study (19) reported that the use of beta-blockers could mitigate improvements in aerobic capacity in healthy subjects. In the present study, there were more patients using beta-blockers in the CET group than in the IET group. However, our results suggest that beta-blockers had no effect on the increase in aerobic capacity after exercise training in CAD patients. In the same context, Vanhees et al. (20) observed similar increases in peak oxygen uptake in CAD patients with and without beta blockers (36 and 34.5%, respectively).

CET and IET showed a similar post-intervention ventilatory efficiency response, as shown by a decrease in VE/VCO_2_ Slope. Additionally, both the CET and IET groups demonstrated an increase in ΔPetCO_2_ at rest after the exercise intervention. Based on these findings, our results suggest that the increase in ventilatory efficiency after aerobic exercise training could be related to a reduction in ventilation/perfusion mismatch (21). Furthermore, it has been suggested that increases in ventilatory efficiency after aerobic exercise training may be related to attenuation of hypercapnic CO_2_ chemosensitivity (22). However, in spite of these previous findings, we failed to observe a correlation between VE/VCO_2_ Slope and OUES after the exercise intervention in either group. In fact, the present results suggest that the improvement in OUES after the interventions may not be associated with the physiological mechanisms related to the decreased VE/VCO_2_ Slope in CAD patients.

A limitation of this study is the fact that there were more hypertensive patients in the CET group than in the IET group. In general, hypertensive patients exhibit a greater increase in blood pressure during exercise than non-hypertensive subjects of the same age (23,24). The higher systemic peripheral resistance associated with hypertension at all levels of exercise may result in increased left ventricular workload and consequently an inadequate cardiac response during exercise.

In conclusion, our findings demonstrate that both CET and IET resulted in similar improvements in OUES in patients with CAD. These data suggest that improvements in OUES in CAD patients after aerobic exercise training may be dependent on both peripheral and central mechanisms.
